# Contribution of LFP dynamics to single-neuron spiking variability in motor cortex during movement execution

**DOI:** 10.3389/fnsys.2015.00089

**Published:** 2015-06-22

**Authors:** Michael E. Rule, Carlos Vargas-Irwin, John P. Donoghue, Wilson Truccolo

**Affiliations:** ^1^Department of Neuroscience, Brown UniversityProvidence, RI, USA; ^2^Institute for Brain Science, Brown UniversityProvidence, RI, USA; ^3^Department of Veterans Affairs, Center for Neurorestoration and Neurotechnology, Brown UniversityProvidence, RI, USA

**Keywords:** neural dynamics, neural point processes, generalized linear models, local field potentials, neural variability

## Abstract

Understanding the sources of variability in single-neuron spiking responses is an important open problem for the theory of neural coding. This variability is thought to result primarily from spontaneous collective dynamics in neuronal networks. Here, we investigate how well collective dynamics reflected in motor cortex local field potentials (LFPs) can account for spiking variability during motor behavior. Neural activity was recorded via microelectrode arrays implanted in ventral and dorsal premotor and primary motor cortices of non-human primates performing naturalistic 3-D reaching and grasping actions. Point process models were used to quantify how well LFP features accounted for spiking variability not explained by the measured 3-D reach and grasp kinematics. LFP features included the instantaneous magnitude, phase and analytic-signal components of narrow band-pass filtered (δ,θ,α,β) LFPs, and analytic signal and amplitude envelope features in higher-frequency bands. Multiband LFP features predicted single-neuron spiking (1ms resolution) with substantial accuracy as assessed via ROC analysis. Notably, however, models including both LFP and kinematics features displayed marginal improvement over kinematics-only models. Furthermore, the small predictive information added by LFP features to kinematic models was redundant to information available in fast-timescale (<100 ms) spiking history. Overall, information in multiband LFP features, although predictive of single-neuron spiking during movement execution, was redundant to information available in movement parameters and spiking history. Our findings suggest that, during movement execution, collective dynamics reflected in motor cortex LFPs primarily relate to sensorimotor processes directly controlling movement output, adding little explanatory power to variability not accounted by movement parameters.

## Introduction

The variability of neuronal responses at the level of single-neuron spiking is a fundamental problem in neuroscience (Shadlen and Newsome, 1998; Churchland, 2010; Churchland and Abbott, 2012). Neuronal responses in neocortex to repeated stimuli presentation or behavioral tasks show substantial variability. Determining the sources of this variability is particularly important for understanding encoding of stimuli and behavioral parameters in neuronal ensembles. The issue is also critical for the development of reliable brain machine interfaces for the restoration of movement, communication, and sensory function in people with sensorimotor impairments (e.g., Hochberg et al., 2012). Beyond intrinsic stochasticity due to, for example, thermal noise and synaptic release failure (Faisal et al., 2008), variability in cortical neural responses has been proposed to arise from fluctuations in spontaneous, ongoing neural dynamics (Arieli et al., 1996; Wörgötter et al., 1998; Truccolo et al., 2002; Carandini, 2004). Although often neglected, spontaneous and ongoing neural dynamics are likely to affect how neurons respond to sensory inputs or even how they modulate their activity during behavior. In this way, spontaneous neural dynamics can provide a background of contextual effects which otherwise may appear as spiking variability (Fiser et al., 2004; Hermes et al., 2012; Goris et al., 2014) due to noise.

Local field potential (LFP) oscillations in different frequency bands result, to a large extent, from ongoing collective dynamics, i.e., modes of coordinated or coherent activity in neuronal populations (Nunez and Srinivasan, 2006; Buzsáki et al., 2012). Previous studies have investigated how features in multiband LFP oscillations relate to sensory stimuli and behavior and how decoding based on LFP features compares to decoding based on spiking activity (Belitski et al., 2008; Bansal et al., 2012). Additionally, some studies have examined how well LFP features predict spiking activity (e.g., Haslinger et al., 2006; Montemurro et al., 2008; Rasch et al., 2008; Kayser et al., 2009; Kelly et al., 2010). However, most of these studies have focused on early sensory cortices and most have been conducted during anesthesia, a neural state distinct from alert and active behavior. More importantly, none of the above studies have addressed how well ongoing collective dynamics reflected in LFPs account for single-neuron spiking variability that is not explained by behavioral parameters (e.g., movement kinematics). For example, the result in Bansal et al. (2012) showing LFP and spiking activity are redundant with respect to decoding kinematics does not address the issue of excess variability in single-neuron spiking.

Here, we examined and quantified how well features in multiband LFP oscillations account for single-neuron variability not explained by behavioral parameters in a naturalistic 3-D reach and grasp task performed by rhesus macaques (*Macaca mulatta*). The behavioral task elicited diverse reaching and grasping kinematics, and included reaching to grasp different objects with different styles of grip.

We examine various LFP features, such as the amplitude envelope and phase, of several LFP bands. The frequency bands included low (<2 Hz; delta) frequency components that are common in this task, including primarily motor related responses associated with these continuously performed sequences of reach and grasp actions (Bansal et al., 2011). In addition, these low frequency signals tend to be highly correlated to the neuronal population spike count (e.g., Bansal et al., 2012, Figure 1). Other bands included the theta (2–7 Hz), alpha (7–15 Hz), beta (15–30 Hz), gamma (30–60 Hz), high gamma (60–100 Hz), and higher frequency bands (100–200 Hz and 200–400 Hz). Although beta band oscillations dominate motor cortex LFP activity during movement preparation (Jasper and Penfield, 1949; Murthy and Fetz, 1992; Sanes and Donoghue, 1993; Brovelli et al., 2004; Rubino et al., 2006), they are also characteristic during execution of isometric force tasks and other steady state conditions (Baker et al., 2001) and, less frequently, during the execution of reach and grasp actions (Reimer and Hatsopoulos, 2010). Neocortical LFP activity in higher (>100 Hz) frequency bands is generally considered to reflect fluctuations in multiunit activity resulting from coordinated activity in neuronal populations (Zhuang et al., 2010; Bansal et al., 2012; Buzsáki et al., 2012; Scheffer-Teixeira et al., 2013), and thus is also a reflection of ongoing collective dynamics.

**Figure 1:**
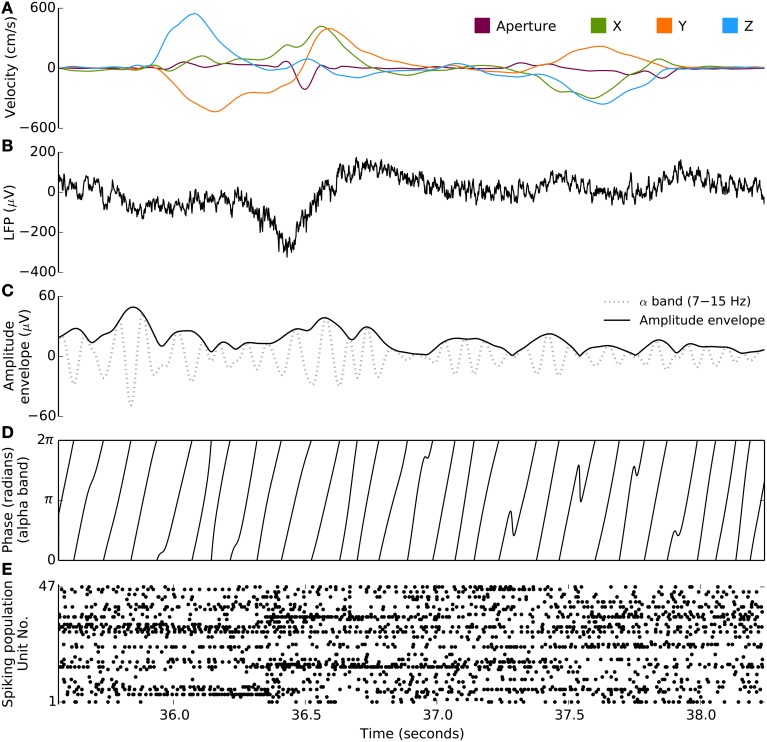
**Behavioral and neural signals during the free-reach and grasp task**. Kinematic, spiking, and LFP data from a single trial of the free reach to grasp task (monkey S, area M1). **(A)** Velocity of 3D wrist endpoint (“‘X,” “Y,” “Z”) and distance between the thumb and forefinger (“Aperture”). **(B)** Broadband LFP, low-pass filtered at 500 Hz during two reach and grasp movements. **(C)** Instantaneous amplitude envelope (bold trace), via Hilbert transform, from the bandpass filtered LFP (gray trace). This particular example is filtered in the 7–15 Hz range. **(D)** Corresponding instantaneous phase extracted from the same bandpass filtered signal as in **(C)**. **(E)** Spiking population raster during this trial: spikes from 47 units are plotted along the y axis. Neurons show different task-related modulations.

We quantified the amount of variability accounted for by LFP features by fitting point process models (Truccolo et al., 2005, 2010) in which the conditional intensity function (“instantaneous” conditional spiking rate) was modeled as a function of covariates, including the ongoing LFP features mentioned above. To assess the amount of spiking variability explained by various models, we compare the relative predictive power of LFP features, reach and grasp kinematics, intrinsic spiking history, and combinations of these covariates, using receiver operating characteristic (ROC) curve analysis.

## Methods

### Behavioral task

Data from three male rhesus macaques were examined in this study, C, R, and S. Data from C have been used previously in Vargas-Irwin et al. (2010). All experimental procedures were conducted as approved by the local Institutional Animal Care and Use Committee (IACUC). This study employed a task termed Free-Reach and Grasp (FRG). In FRG, an experimenter pseudo-randomly swings within the monkey's reach one of various small (3–5 cm) objects of differing shapes attached to a string, and the monkey is rewarded for grasping the object. The FRG task was designed to elicit naturalistic and continuous three-dimensional reach and grasp behaviors that require online motor control (Vargas-Irwin et al., 2010). Reach and grasp actions were organized in blocks within each experimental session. We analyze data from the entire FRG block, and so our data contains a diversity of behavioral conditions, including visually guided reaching (including online corrections as the objects' movement is unpredictable), the grasping and holding of the object, and the period of juice reward (see Vargas-Irwin et al., 2010).

### Kinematic feature extraction

Arm and hand kinematics were recorded at 240 frames per second using a Vicon motion capture system (Vicon Motion Systems; Oxford Metrics Group) as detailed in Vargas-Irwin et al. (2010). This system employs infrared-reflective markers to track arm and hand positions in real time, and is capable of inferring missing data from briefly occluded markers. For our analysis, we focus on the 3-D kinematics measured at the wrist, as well as the distance between the thumb and forefinger (grasp aperture), as indicators of the kinematics related to reaching (i.e., hand position in space) and grasping. We analyzed kinematics features similar to those used in Hatsopoulos et al. (2007). These are normalized velocity trajectories of both the wrist endpoint and grip aperture in time, combined with zero lag position and speed. For comparison, we also analyze position trajectories of these markers over time. The velocities of motion capture markers were estimated using a Savitzky-Golay filter generated by fitting a 5th order polynomial to a discrete differentiation operator at the sampling rate of the kinematics. The polynomial extended 25 samples (10 ms) to either side of the current time-point. The polynomial order was selected such that frequencies higher than 20 Hz were attenuated, so that the resultant velocity trajectories were sufficiently smooth to down-sample at 40 samples-per-second. Velocity trajectories were sampled from the smoothed velocity every 25 ms, starting 100 ms before the current time-point and extending 300 ms into the future. Similarly, a smoothed position estimate was extracted using Savitzky-Golay filters based on a 5th order polynomial fit to a discrete impulse. For the normalized velocity feature set, we followed the steps in Hatsopoulos et al. (2007), i.e., velocities were normalized by the L2 norm of the velocity trajectory. 3-D wrist position and grip aperture were normalized separately. The average speed and position over the trajectories were added as additional features. A separate feature set based on position trajectories was used for comparison. Position trajectories were taken from the position estimates, sampled at 40 samples-per-second, covering the same period (100 ms before to 300 ms after) as the normalized velocity trajectories.

### Neural recordings

Recordings were made using microelectrode arrays (Blackrock Microsystems), as previously described in Vargas-Irwin et al. (2010). Electrodes were 1.5 mm long, likely targeting layer 5 of motor cortex. Monkey G was implanted with 4 × 4 mm 96-microelectrode arrays in both M1 and PMv. Two monkeys (R,S) were implanted with one 96-microelectrode array in PMv, and a 3.2 × 2.4 mm 48-microelectrode array in each of M1 and PMd areas. Electric potential signals were recorded broadband (analog band-pass filtered to 0.3 Hz – 7.5 kHz; digitized at 16bit and sampled at 30 kilosamples per second) using a Cerebus Data Acquisition System (Blackrock Microsystems). For spike detection, recorded signals were digitally filtered with a 250 Hz fourth-order high-pass Butterworth filter. For each electrode, candidate spikes (action potentials) were identified online via threshold crossing detection in the amplitude of the high-pass filtered signal (Cerebus Data Acquisition System). Preliminary spike sorting was performed by a custom automated spike sorter (Vargas-Irwin and Donoghue, 2007), and verified using the commercial Plexon Offline Sorter (Plexon Inc.). Candidate units to be included in the analysis had a minimum signal-to-noise ratio (SNR) of 3.0 (defined as in Vargas-Irwin and Donoghue, 2007). In addition, we required that (a) the inter-spike-interval (ISI) histogram display a clear refractory period to exclude potential multi-unit clusters; (b) that there be at least 500 inter-spike-interval events smaller than 100 ms within the task data, to provide accurate estimates of spike-history filters; and (c) that units be clearly separated into different clusters in the PCA feature space. Electrodes exhibiting cross-talk or excessive noise were excluded from analysis. For monkey C, LFPs were extracted during recording sessions from the broadband signal using a causal 500 Hz fourth-order low-pass Butterworth filter, and stored at two kilosamples per second. LFP data from monkeys R and S were filtered offline to match this processing. Channels displaying cross-talk or excessive noise were excluded from the analysis.

### LFP feature extraction

For analysis of band-limited LFP oscillations, LFP signals were filtered using causal (forward only) fourth-order Butterworth low-pass and band-pass filters, with cutoff frequencies 0.3–2 Hz (δ), 2–7 Hz (θ), 7–15 Hz (α), 15–30 Hz (β), 30–60 Hz (γ), 60–100 Hz (high γ), 100–200 Hz (MUA1), 200–400 Hz (MUA2). The 0.3–2 Hz low-pass signal captures slow motor-evoked potentials (MEPs). The two highest-frequency bands are likely to reflect a substantial contribution from multi-unit activity (MUA) to neocortical LFPs, as well as other possible high-frequency source signals. For the narrow delta, theta, alpha, and beta frequency bands, we considered four features: the instantaneous phase and amplitude of the analytic signal, and the real and imaginary component of the analytic signal. The LFP analytic signal was computed from the band-pass filtered LFP using the Hilbert transform. LFP instantaneous phase and amplitude were computed as the complex argument and modulus of the analytic signal, respectively. (The real component of the analytical signal corresponds to the band-pass LFP itself.) For the broader, higher frequency gamma and multi-unit bands, we use only the analytic signal and the amplitude envelope. Feature extraction was performed on the LFP sampled at two kilosamples per second, and decimated to one kilosample per second for neural point process modeling.

### Spike contamination

In this analysis, we predict single-unit spiking from features of the LFP recorded on the same electrode as the isolated unit. Because of this, when predicting neuronal spiking from LFP features, it is important to prevent action potentials from contaminating the filtered LFP. We elected not to use existing spike removal procedures (e.g., Zanos et al., 2012) because the broadband LFP data were unavailable for monkey C, and because spike-removal methods make implicit assumptions about which features of the LFP relate to the spike waveform as opposed to collective dynamics locked to spiking. Instead, we employed causal filtering to extract LFP features. Although spike contamination can occur as low as 10 Hz (Waldert et al., 2013), causal filtering restricts this contamination to times following a spike, avoiding the confound of predicting spikes from themselves (i.e., via their contamination of the LFP features). Although the discrete Hilbert transform used to compute phase and amplitude features is non-causal, the effective filters created by the composition of the Hilbert transform with the causal band-pass filters remain predominantly causal. As a further precaution, and to guard against imprecision in localizing spike times, we added 1 ms delay to LFP features. Under this approach, the noncausal contribution to the imaginary component of the analytic signal was negligible: less than 0.14% of the impulse response (measured as the percentage of the area under the absolute impulse response) was non-causal. Since causal filters can add amplitude and phase distortions, we addressed this concern by comparing the predictive power of causally filtered LFP and that of zero-phase (non-causal) filtered LFP, which contains no delay. We determined that the choice of causal verses zero-phase filtering did not alter the conclusions of this paper for frequencies below 30 Hz. Zero-phase filtering for higher frequencies resulted in higher predictive power in some cases, which was likely the result of action potential contamination as supported by the recent studies mentioned above.

### Intrinsic spiking history

To assess the extent to which a neuron's own spiking history explains spiking variability, and to compare its predictive power to that of kinematic and LFP features, we included features of spiking history in our modeling (Truccolo et al., 2005). In addition to intrinsic biophysical processes (refractory/recovery period, bursting, etc.), spiking history can potentially also reflect indirect neuronal network dynamics effects. For example, spiking history models are capable of capturing spiking rhythmicity that may arise as a result of oscillatory input. We used raised cosine bases in logarithmically scaled time, covering the past 100 ms of spiking activity, to estimate temporal filters (see Pillow et al., 2008 and Truccolo et al., 2010, for more details). The resulting temporal filters were convolved with the past spiking activity to capture history effects on the spiking probability at a given time. Ten basis functions were used. More recent spiking history, typically related to after-spike refractory and recovery periods, and bursting, was modeled with more localized (finer temporal resolution) basis functions. Longer-term history effects can capture intrinsic rhythmicity and also, implicitly, network dynamics.

### Stochastic neural point process models

We used a generalized linear point process model (Truccolo et al., 2005) to explore the extent to which different covariates explain spiking variability. The probability of a neuron spiking in a sufficiently small time interval, indexed by *t*, of duration Δ, can be written as

(1) $$\mathrm{Pr}(Y_{t}=1|\lambda_{t})=\lambda_{t}\Delta +o(\Delta ),$$

where *Y_t_* corresponds to the spiking activity at time *t*, *Y_t_* = 1 for a spike, 0 otherwise, and λ_*t*_ is the conditional intensity function (“instantaneous conditional spiking rate,” in spikes per second) of the modeled neuron. The bin size Δ must be chosen small enough such that the probability of two spikes occurring within the same time bin is negligible. Here Δ = 1 ms. We used a regularized maximum likelihood approach to model the logarithm of the conditional intensity function as a linear combination of model features:

(2) $$\ln(\lambda_{t})=\mu +A\cdot X_{t},$$

where *X_t_* is the covariate vector at time *t*, *A* is a vector of model parameters, and μ is a parameter related to background activity level. *X_t_* can refer to LFP features at time *t*, past and future kinematics, convolutions of intrinsic spiking history up to but not including time *t* with temporal filters, or combinations of these covariates. For example, for a given Hilbert-transform of an LFP band, *z*(*t*), the feature vector

(3) Xt=(|zt|, Re(zt), Im(zt), cos(Arg(z)), sin(Arg(z)))

corresponds to a model with cosine tuning to a preferred Hilbert phase θ_0_, as well as amplitude envelope and analytic signal features, i.e.,

(4) ln(λt)=μ+a1|zt|+a2Re(zt)+a3Im(zt)+a4cos(θ0−Arg(z))

### Model fitting

Model estimation is solved by finding parameters *A* and μ that maximize the L2-regularized log-likelihood of the observed spiking activity (Truccolo et al., 2005):

(5) $$\underset{A,\mu }{argmax}[\ln(\mathrm{Pr}(Y|X,A,\mu ))]=\frac{1}{T}\sum_{t=1}^{T}[Y_{t}\ln(\lambda_{t}\Delta )-\lambda_{t}\Delta ]-\alpha \|A{\|}^{2},$$

where α is a penalty or regularization parameter. The log-likelihood is normalized by the number of samples *T* so that the strength of regularization does not depend on the amount of data. The parameter μ is not penalized. All features are z-scored prior to model fitting to ensure that all features are zero mean and of comparable scale, which ensures that the L2 penalty is applied equally to all features and improves numerical accuracy.

We used a gradient descent approach for the minimization of the negative log-likelihood under L2 regularization. Models were fit under a two-tier cross-validation scheme. An outer level of 10-fold cross-validation ensures that results are not overfit. An inner level of cross-validation selects the regularization parameter α. Ten values of the regularization parameter α, base-10 logarithmically spaced between 1e-9 and 1e2 inclusive, as well as α = 0, were tested. On each of the 10 outer-level cross-validations, 90% of the data were taken as training data, and 10% were reserved for testing. The training data were split randomly into two equal groups. For each group, models were generated for each value of the regularization parameter α. The value of the regularization parameter that led to the best generalization (in terms of predictive power, see below) in this internal cross-validation step was selected for fitting a model on all of the training data. This model was then validated on the remaining 10% of the data that had been withheld for testing. This two-tier cross-validation procedure was repeated 10 times, such that all of the available data was used for model validation and assessing predictive power. To confirm that L2 regularization sufficiently prevented over-fitting when adding LFP features to the kinematics model, we shuffled LFP features in 100 ms blocks with respect to the spiking activity. We found that adding these non-informative features to the kinematics and kinematics-history combined models reduces the predictive power very little, by at most 0.03, and with the population mean decrease ranging from 0.001 and 0.006 across all sessions. This difference is too small to alter the conclusions of our study. In preliminary analysis, we also explored L1 regularization and also a mixed L1-L2 regularization (fitted via elastic net, Friedman et al., 2010). We found that L2 regularization outperformed these alternatives, both in terms of computational time and predictive power under generalization. Additionally, we found that L2 regularized GLM models outperformed simpler approaches, such as naive Bayes and spike-triggered average and covariance analysis.

### Assessment of predictive power

Model performance was evaluated using the area under the ROC convex hull (AUC) measured on testing data, using the model (Equation 2) to compute the conditional spiking probability, Pr(*Y_t_* = 1|*X_t_*, *A*, μ) ≈ λ_*t*_(*X_t_*, *A*, μ)Δ, from observed covariates (Truccolo et al., 2010). ROC analysis assesses predictive power in the context of binary (in this case spike train) sequences (Fawcet, 2006). We report predictive power (PP) as 2×*AUC*−1, which ranges from 0 (no predictive power) to 1 (complete prediction of single-neuron spikes in 1 ms time bins). Note that this predictive power measure is based on both true and false positive rates, since is derived from the ROC analysis.

## Results

We are interested in how well collective neural dynamics, as reflected by features in ongoing and evoked multi-band LFP signals, can explain motor cortex single-neuron spiking variability not accounted for by motor behavioral covariates such as reach and grasp kinematics. We first demonstrate that the examined LFP features can predict single-neuron spiking in motor cortex, then we assess the extent to which this predictive power compares and is redundant to information available in 3-D kinematics. We also assess the extent to which intrinsic spiking history, i.e., temporal dynamics or correlation in the modeled spiking activity itself, adds predictive power to kinematics, and evaluate whether LFP features remain predictive conditioned on kinematics and intrinsic spiking history.

Datasets from seven experimental sessions were used in these analyses: two each from monkey C and R, and three from monkey S. Sessions from monkeys R and S were collected within a week of each-other. The two sessions from monkey C were collected 3 months apart. Between three and nine reach and grasp blocks, averaging 140 s long, were collected on each session. Each session included 15–42 successful free-reach-to-grasp trials or reaches in each block. This yielded 7–17 min of FRG task data, averaging 10 min of data per session. A detailed statistical description of the kinematics and examples of kinematic trajectories in these experimental blocks can be found in Vargas-Irwin et al. (2010) and Bansal et al. (2012), who reported some of the data from monkey C in this task. An example of kinematics trajectories with the corresponding neuronal ensemble spike raster is shown in Figure 1. For each array in each session, between 19 and 83 well-isolated units were identified for analysis (mean = 52, σ = 16). For a given monkey and area, some of the neurons are thought to be the same across sessions, for this reason we do not combine sessions when we perform statistical significance tests.

### Features of LFP oscillations predict single-neuron spiking with substantial power

First, we evaluated the ability for multiband LFP features to predict single-neuron spiking. We fit a regularized generalized linear model to predict single-unit spiking (1 ms time resolution) from multiband LFP features. Spiking probability at any given 1 ms time interval was modeled as a function (Equations 1–2, Methods) of features of ongoing LFP activity. LFP features included instantaneous phase and amplitude envelope as well as the analytic signal, extracted via a Hilbert transform, from four narrow LFP bands (Methods), δ (0.3–2 Hz, motor related potentials), θ (2–7 Hz), α (7–15 Hz), β (15–30 Hz), as well as the amplitude envelop and analytic signal for four broader, higher frequency bands: γ_1_ (30–60 Hz), γ_2_ (60–100 Hz), and two multi-unit activity (MUA) bands MUA_1_ (100–200 Hz), and MUA_2_ (200–400 Hz). Figure 1 illustrates the signal processing steps involved in the computation of the instantaneous phase and magnitude for a single frequency band.

**Figure 2:**
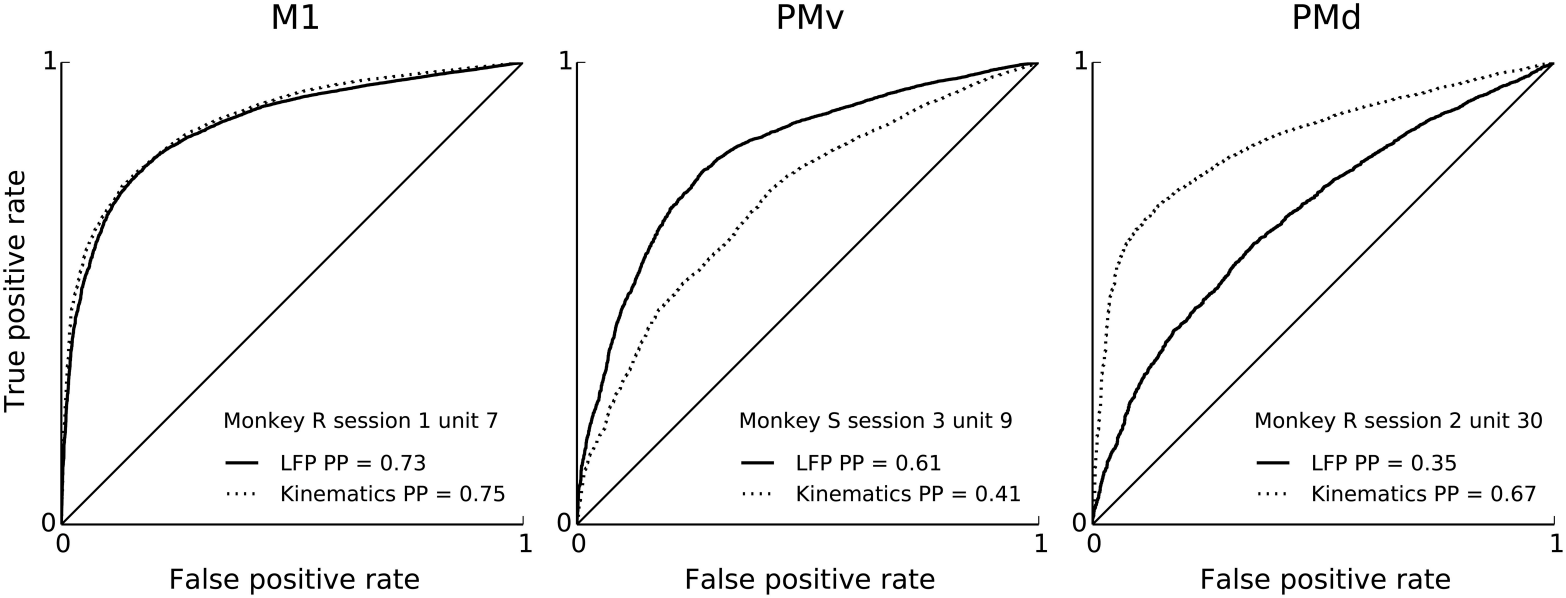
**Features of ongoing and evoked multiband LFP oscillations predict single-neuron spiking: examples**. ROC curves (solid) for neural point process models based on multiband LFP features (Methods: LFP feature extraction). Examples correspond to different neurons, areas, monkeys and sessions. From left to right: M1, PMv, and PMd. For comparison, ROC curves (dashed) corresponding to models based on kinematics features are shown. Both LFP features and kinematic features achieve substantial single-unit spiking predictive power. LFP features include the instantaneous phase, amplitude envelope, and analytic signal, in the δ = 0.3–2 Hz, θ = 2–7 Hz, α = 7–15 Hz, and β = 15–30 Hz LFP bands, as well as amplitude envelope and analytic signal in the γ_1_ = 30–60 Hz, γ_2_ = 60–100 Hz, MUA_1_ = 100–200 Hz, and MUA_2_ = 200–400 Hz bands (see Methods: LFP feature extraction). Predictive power is the area under the ROC curve normalized, i.e., 2 × AUC –1, such that 0 is chance level and 1 is perfect prediction. Predictive power was evaluated under 10-fold cross-validation.

**Figure 3:**
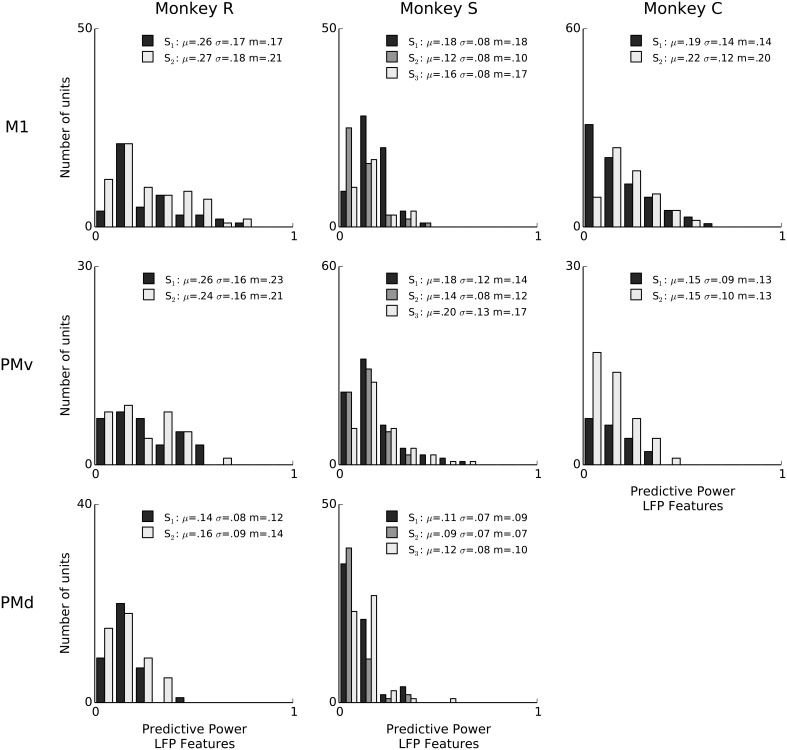
**Features of ongoing and evoked multiband LFP oscillations predict single-neuron spiking: summary across animals and areas**. Histogram counts of the predictive power of point-process models based on LFP features, for all isolated units. LFP features include the instantaneous phase, amplitude envelope, and analytic signal, in the δ = 0.3–2 Hz, θ = 2–7 Hz, α = 7–15 Hz, and β = 15–30 Hz LFP bands, as well as amplitude envelope and analytic signal in the γ_1_ = 30–60 Hz, γ_2_ = 60–100 Hz, MUA_1_ = 100–200 Hz, and MUA_2_ = 200–400 Hz bands (see Methods: LFP feature extraction). LFP was consistently predictive of spiking variability for a subset of neurons in all sessions. In the figure legends, “S” indicates the session, μ is the mean of each distribution, *m* is the median, and σ is standard deviation. Sessions have different numbers of units, and the differences in bar height also reflect differences in sample size (e.g., monkey C area PMv).

We report the extent to which a model explains spiking variability in terms of “predictive power” (PP). Predictive power is the normalized area under the receiver operator characteristic (ROC) curve such that 0 corresponds to chance level and 1 to perfect prediction of spike times at 1ms resolution (see Methods: Model fitting; Methods: Assessment of predictive power). Figure 2 shows PPs obtained from three example neurons from different monkeys and areas. For illustration and comparison, we also show the corresponding PPs based on a model including only kinematics features related to lagged 3-D velocity and position (similar to “pathlets” in Hatsopoulos et al., 2007) and grasp aperture (Methods). The examples show a case (Figure 2, left) in which LFP features and kinematics both explained a substantial fraction of spiking variability (PP = 0.73, 0.75, respectively) and two other examples in which LFP features did better and worse than kinematics, respectively. Overall, we found that LFP features were typically predictive, and that for some neurons LFP accounted for a substantial (i.e., PP > 0.5) fraction of variability. We performed a permutation test to assess chance level LFP predictive power by shuffling LFP features in 100 ms blocks relative to spiking, and found that the 95% chance predictive power ranged from 0.03 to 0.06 across sessions. Across sessions and areas, between 85% and 100% of units showed LFP predictive power higher than this chance level. As shown in Figure 3, high predictive power from LFP was consistent across all monkeys, motor cortical areas (PMv, PMd, and M1) and sessions. This finding demonstrates that collective dynamics reflected in ongoing and evoked LFP oscillations can account for a substantial fraction of single-neuron spiking variability.

### LFP features contributing to prediction of single-neuron spiking

We examined whether some of the multiband LFP features contribute more to prediction of single neuron spiking than others. Analysis based on estimated model coefficients is complicated because of the nonlinear (multiplicative) interactions between different features (amplitude, phase, or analytical signal) in different frequency bands. Instead, we performed an analysis based on fitting a single model for each feature separately and assessing how well each separate model and feature predicted spiking. This allowed easy visualization of the predictive power of each individual LFP feature.

**Figure 4:**
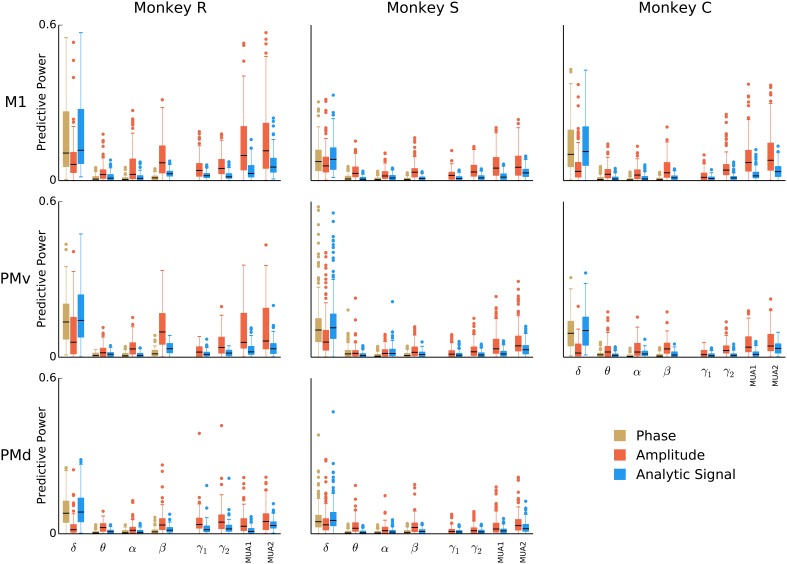
**Breakdown of LFP predictive power by frequency band and LFP feature**. Box-plots over the population of isolated units (all sessions combined) showing the predictive power of models based on phase, amplitude, or analytic signal features in isolation from each of eight bands. To better assess the individual predictive power of each LFP feature, models were fitted for each feature separately. Certain features, such as the instantaneous phase and analytic signal for the 0.3–2 Hz band, as well as the analytic signal amplitude modulation above 100 Hz, consistently predict spiking across all animals and areas.

This analysis revealed some trends common to all animals and motor cortex areas, but also some variations (Figure 4). Consistently across animals and areas, low-frequency local field potentials (δ, 0.3–2 Hz) showed predictive power in the time domain signals and phases, but not amplitude envelopes. Additionally, the amplitude envelope in the multi-unit (100–200 and 200–400 Hz) bands was predictive, more-so than the signal. The low-frequency <2 Hz analytic signal was the most predictive for 49% of units (486 units out of 991), and the instantaneous amplitude envelope and phase the most predictive for 13% and 8% of units (133 and 82 units out of 991), respectively. The amplitude envelope in the 200–400 Hz band was the most predictive for 14% of units (142 units out of 991). Features from intermediate 2–100 Hz bands generally performed poorly, with the exception of beta (15–30 Hz) amplitude, which although less predictive than the aforementioned features, was still amongst the top 4 most predictive features for 23% of units (227 units out of 991). The finding that LFP amplitude was predictive for the beta-frequency LFP was strongest in monkey R for areas M1 and PMv. To understand in more detail how the predictive power in beta amplitude varies across monkeys and areas, we examined the distribution of the model parameter weights for beta amplitude (in the case of the amplitude-only model, the parameter matrix *A* in Equation (2) is simply a single scalar parameter). Model weights for beta amplitude in monkey R areas M1 and PMv were more negative (mean ± 2SD for M1 and PMv were −0.18 ± 0.4 and −0.23 ± 0.4) than those from other monkeys and areas (−0.04 ± 0.16), indicating that a reduction in beta amplitude is typically associated with an increase in firing rate.

### Predictive power of kinematics during naturalistic reach and grasp movements

**Figure 5:**
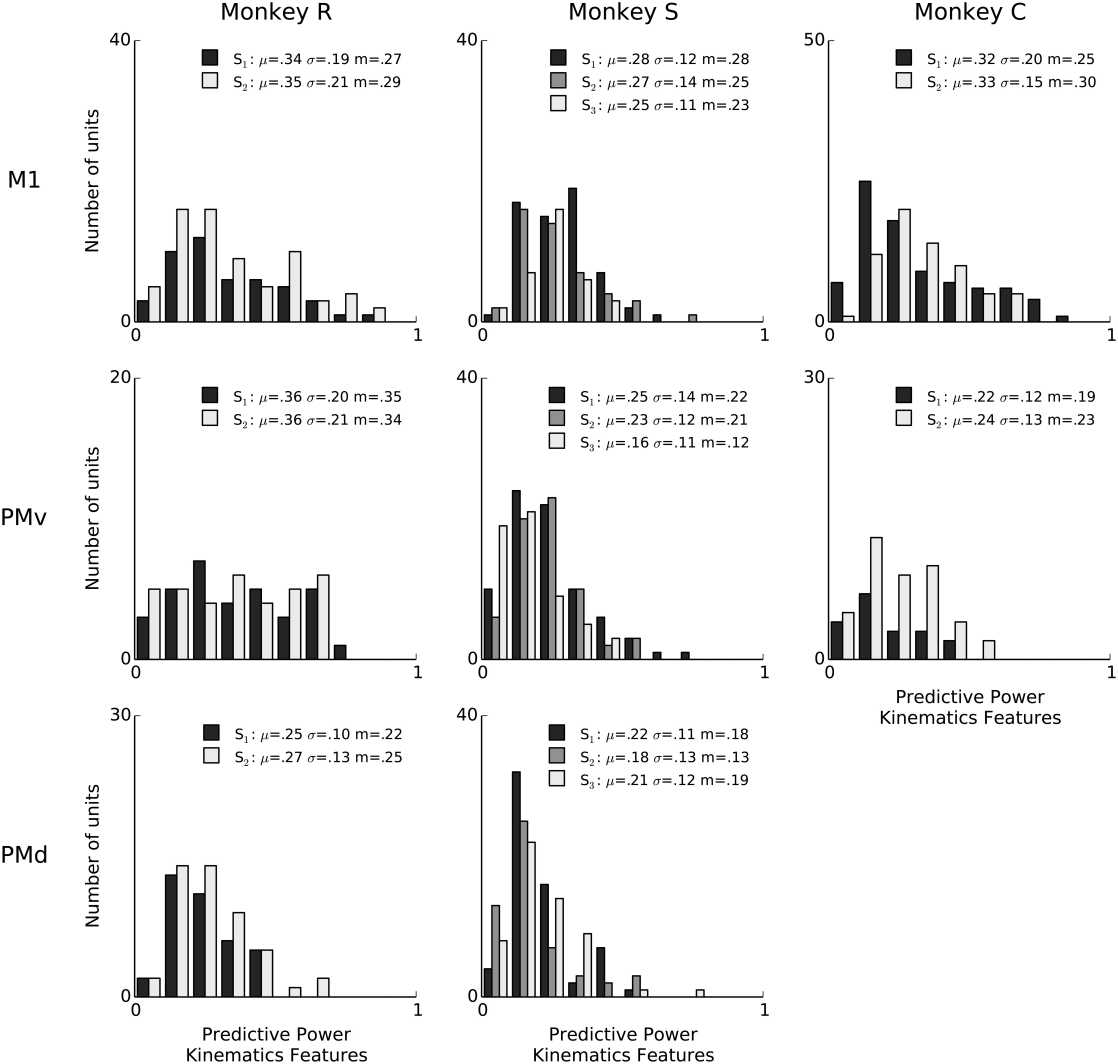
**Kinematic features predictive power for single-neuron spiking: summary across animals and areas**. Histogram counts of the predictive power of point-process models based on kinematics features, for all isolated units. Kinematics features are normalized velocity trajectories of wrist endpoint and grip aperture, extending from 100 ms in the past to 300 ms in the future, sampled every 25 ms, as well as the average speed and zero-lag position for wrist endpoint and grip aperture (Methods: Kinematic feature extraction). In the figure legends, “S” indicates the session, μ is the mean of each distribution, *m* is the median, and σ is standard deviation. Sessions have different numbers of units, and the differences in bar height also reflect differences in sample size (e.g., monkey C area PMv).

We next quantified the predictive power of motor behavior, specifically kinematic features of the 3-D reach and grasp movements. We found that kinematics trajectories can also predict single-neuron spiking with substantial accuracy, at times achieving predictive power levels around 0.8 (e.g., Figure 5, monkey R area M1). However, similarly to LFP features, there was considerable diversity in the extent to which kinematics predicted spiking, with some units being predicted poorly. Similar results were obtained by using position trajectories (i.e., position at multiple time lags with respect to spiking; Methods). The 95% chance level predictive power for kinematics ranged from 0.03 to 0.08, as assessed by shuffling kinematics features in 100ms blocks relative to spiking. Across sessions and areas, between 49 and 100% of units showed LFP predictive power higher than this chance level. These effects were consistent across all animals, sessions, and motor areas (Figure 5), with mean predictive power ranging between 0.16 to 0.36 across sessions and areas. The fact that the task kinematics predict single-unit spiking variability confirms that we are recording from motor cortex populations that exhibit task-modulation and tuning to motor output.

**Figure 6:**
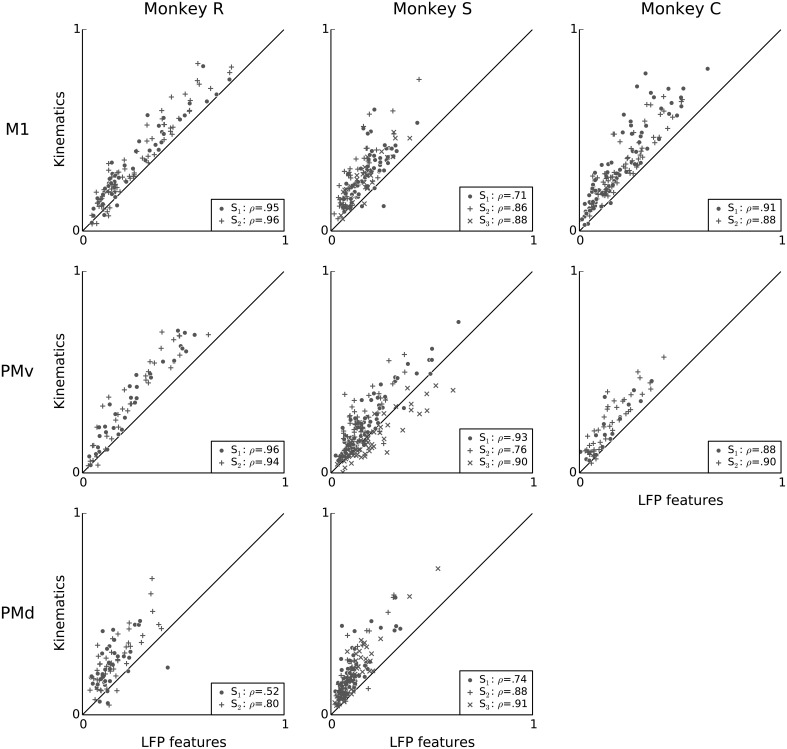
**Kinematics and features of ongoing and evoked multiband LFP oscillations achieve similar predictive power on a neuron by neuron basis**. Scatter plots compare the predictive power of LFP features (x-axis) to that of kinematic features (y-axis). LFP features include the instantaneous phase, amplitude envelope, and analytic signal, in the δ = 0.3–2 Hz, θ = 2–7 Hz, α = 7–15 Hz, and β = 15–30 Hz LFP bands, as well as amplitude envelope and analytic signal in the γ_1_ = 30–60 Hz, γ_2_ = 60–100 Hz, MUA_1_ = 100–200 Hz, and MUA_2_ = 200–400 Hz bands (see Methods: LFP feature extraction). Each data-point is a single unit from one session. The dashed line indicates equality. Most units lie above the dashed line, indicating that kinematic features better predict single-unit spiking variability. In the figure legends, “S” indicates the session, ρ is the Pearson correlation coefficient between the predictive power for kinematics and LFP features. Predictive power from both LFP and kinematics are highly correlated, i.e., neurons that are well predicted by LFP features tend also to be well predicted by kinematics.

Figure 6 directly compares the predictive power of LFP and kinematics features. Overall, the predictive power of LFP features was typically less than that of kinematics during this free reach and grasp task: the difference between the predictive power of models based on kinematics and LFP features ranged from −0.20 to 0.45. With exception of monkey S area PMv, units for which LFP features explain more variability than kinematics are rare. The mean difference in predictive power within each session ranged from −0.04 to 0.14, and the median difference from −0.02 to 0.12, with all (session, area) pairs except monkey S area PMv session 3 displaying significantly better median predictive power for kinematics. (Wilcoxon signed-rank test with *p* < 0.05, corrected for multiple comparisons using Bonferroni correction for 19 (session, area) pairs.) Furthermore, across all monkeys and areas, predictive power of LFP was highly correlated with that of kinematics: the Pearson correlation coefficient between the predictive power of kinematics and LFP features ranged from 0.52 to 0.96, with a mean of 0.86 and a median of 0.88. This raises the possibility that LFP and kinematics shared some common effect, which we address below.

### LFP features are mostly redundant to kinematics when explaining single-neuron spiking variability

In order to determine whether LFP features can account for single-neuron spiking variability not explained by kinematics, we asked whether the predictive information carried in the examined LFP features about single-neuron spiking variability was redundant to the predictive information carried in kinematic features. To assess redundancy we compare the predictive power of point process models based only on kinematics features to models that included both kinematics and LFP features. We used L2 regularization to control for overfitting to the training data due to the larger number of parameters in the models that combined both kinematics and LFP features (Methods). We found that forgoing L2 regularization led to overfitting, in which the larger number of parameters in the combined LFP-kinematics model generalized less well to the evaluation data. Tests using shuffled LFP features confirmed that the L2 regularization approach adequately prevented overfitting (Methods).

**Figure 7:**
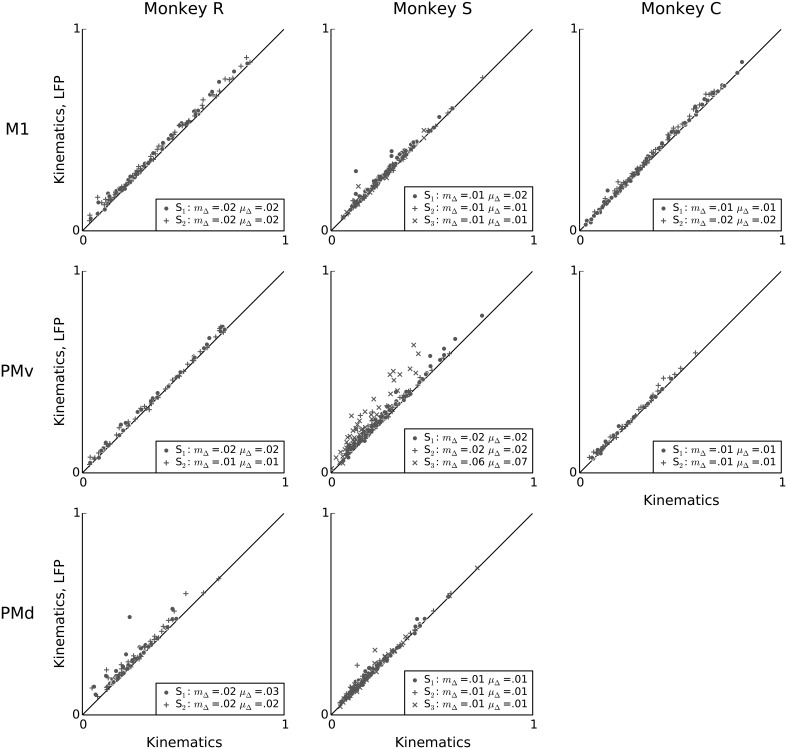
**LFP features add little predictive power to a kinematics model**. Scatter plots comparing, on a unit-by-unit basis, the predictive power of the kinematics model (x-axis), to the predictive power of a model that uses both LFP and kinematics features (y-axis). Although LFP features by themselves can achieve high predictive power for single-neuron spiking (Figure 3), their combination with kinematics typically results in only a small increase in predictive power, suggesting that most predictive information in the examined LFP features is redundant to predictive information in kinematics features. In the figure legends, “S” indicates the session, μ_Δ_ is the mean change in predictive power, and *m*_Δ_ is the median change in predictive power.

Figure 7 compares, on a unit-by-unit basis, the relative predictive powers of kinematics and LFP features. The analysis reveals that, with a few exceptions (e.g., some neurons in PMv in monkey S), LFP features added little predictive power to kinematics. This finding suggests that although LFP features were able to account for a substantial fraction of spiking variability, this information was highly redundant to information available in the examined kinematics features. This finding confirms the conjecture raised earlier, based on the high correlation between LFP and kinematics predictive power (Figure 6), that the information available in these two signals was redundant in terms of prediction of single-neuron spiking activity. Nevertheless, for each session, after adding LFP features, the mean change in predictive power was positive, ranging from 0.008 to 0.07, and the median change in predictive power ranged from 0.006 to 0.06. This median increase was statistically significant for all sessions. (Wilcoxon signed-rank test, *p* < 0.05 corrected for multiple comparisons using Bonferroni correction for 19 (session, area) pairs).

### Intrinsic spiking history adds substantial power to kinematics in the prediction of single-neuron spiking variability

The above findings suggest that the examined features of LFP collective dynamics during movement reflect primarily sensorimotor processes related to motor representations and computations associated with the measured reach and grasp kinematics. Nevertheless, these LFP features accounted for a small, but statistically significant, fraction of neural variability not accounted by the examined kinematics features. To investigate the potential sources of this additional predictive power, we take a detour in this section and consider first the predictive power of a neuron's own spiking history. Here we focused on the preceding 100 ms spiking history, which can capture fast intrinsic biophysical processes such as refractory and recovery periods after an action potential, and also bursting dynamics, which are common in certain types of motor cortex neurons (Chen and Fetz, 2005). In addition, temporal autocorrelations within single-neuron's spiking activity can be induced, for example, by both intrinsic rhythmicity and rhythmicity due to ongoing neuronal network dynamics affecting spiking. We used temporal filters to capture the effects of intrinsic spiking history. Temporal filters were estimated with semi-parametric models using raised cosine functions (Pillow et al., 2008; Truccolo et al., 2010; Methods). Ten logarithmically-spaced raised cosine functions on the past 100 ms were used.

**Figure 8:**
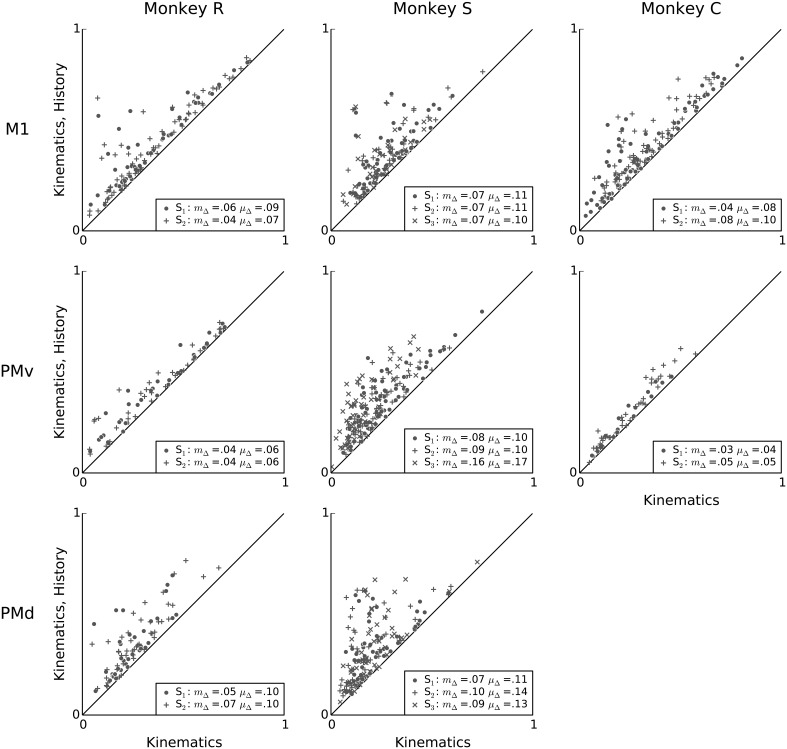
**Intrinsic spiking history carries complementary information to kinematics features**. Scatter-plots showing a unit-by-unit comparison of the predictive power of the kinematics model (x-axis) to that of a model that uses both kinematics features and intrinsic spiking history features (y-axis). Adding 100 ms of intrinsic spiking history information improves prediction substantially for almost all units, consistently across animals and areas. In the figure legends, “S” indicates the session, μ_Δ_ is the mean change in predictive power, and *m*_Δ_ is the median change in predictive power.

When information about a single neuron's own spiking history is added to the model, there is a significant improvement in predictive power compared to a model including only kinematics features (Figure 8). Within each session and area, the mean increase in predictive power ranged from 0.04 to 0.17. The median increase in predictive power ranged from 0.03 to 0.16, and was statistically significant for all sessions and motor areas (Wilcoxon signed-rank test, *p* < 0.05 with Bonferroni correction for 19 (session, array) multiple tests). This result demonstrates that fast-timescale spiking history can explain variability in single-neuron spiking that is not redundant to variability examined by the kinematic features in this motor task.

### Conditioned on spiking history, contribution of LFP features to kinematic models is further reduced

**Figure 9:**
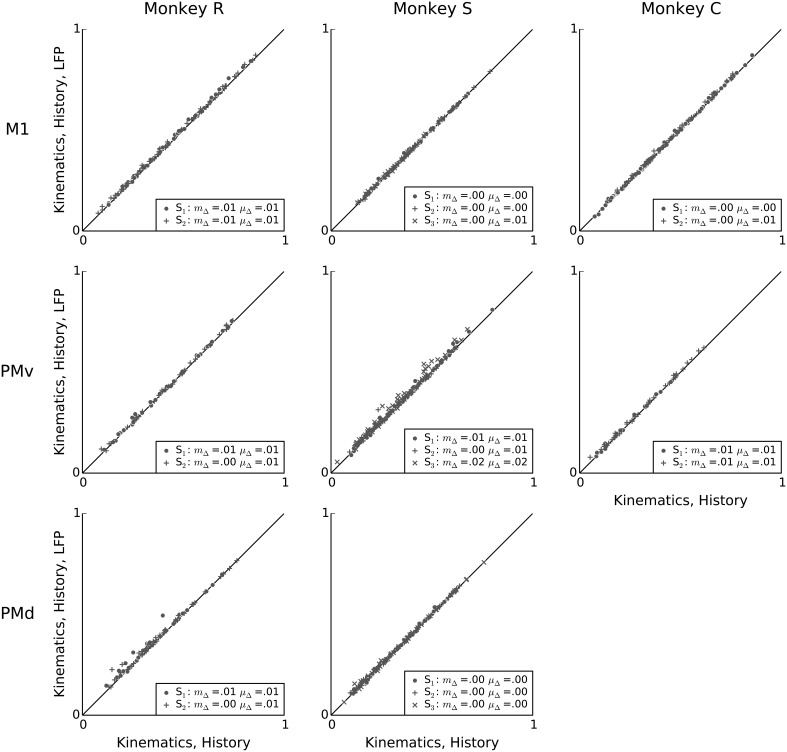
**Conditioned on intrinsic spiking history, the contribution of LFP features to kinematic models is redundant**. Scatter plots comparing the predictive power of a model based on kinematics and intrinsic spiking history (x-axis) to that of a model based on kinematics, intrinsic history, and LFP features (y-axis). LFP features add negligible predictive power after accounting for behavioral and intrinsic spiking history effects. In the figure legends, “S” indicates the session, μ_Δ_ is the mean change in predictive power, and *m*_Δ_ is the median change in predictive power.

Having demonstrated that intrinsic spiking history adds predictive power to kinematic models, we finally assessed whether LFP features can account for variability in single-neuron spiking not accounted for by kinematics and intrinsic history features. Figure 9 shows that, across monkeys and motor areas, adding LFP features to models based on kinematics and intrinsic spiking history leads to no substantial improvement in predictive power. Across sessions and areas, the mean change in predictive power when adding LFP features to a model containing both kinematics features and intrinsic spiking history features ranged from 0.002 to 0.02, and the median change ranged from 0.001 to 0.02. Nevertheless, this median improvement was statistically significant for all but one session (monkey C area PMv session 1) (Wilcoxon signed-rank test, *p* < 0.05 with Bonferroni correction for 19 (session, array) multiple tests).

This result demonstrates that the LFP predictive power not redundant to kinematics features was primarily redundant to information available in the recent 100 ms spiking history in this motor task. In other words, additional single-neuron variability not explained by kinematics seems to be better explained by fast-timescale features in intrinsic spiking history than by the examined motor cortex LFP features in this reach and grasp task.

## Discussion

Neocortical neurons are embedded in large networks possessing highly recurrent connectivity. Recurrent connectivity typically leads to rich spontaneous collective dynamics. The extent to which these spontaneous dynamics contribute to single neuron variability in awake behaving primates, and how these dynamics interact with sensory inputs and behavioral outputs is an important open question in neuroscience. Here we examined this problem in the context of collective dynamics reflected in LFP oscillations at multiple frequencies in three different areas of motor cortex in monkeys performing naturalistic 3-D reach and grasp actions. These LFPs are thought to result, to a large extent, from collective modes of activity driving spatially coherent postsynaptic potentials at multiple spatiotemporal scales (Nunez and Srinivasan, 2006; Buzsáki et al., 2012). LFP features (e.g., amplitude envelope, phase, and analytic signal) in eight different frequency bands predicted single neuron spiking (1 ms time resolution) with significant predictive power for many neurons in all of the three examined motor cortex areas (PMv, PMd, and M1). Neurons for which LFP predictive power was high tended also to show high kinematics predictive power. In fact, this relationship was close to linear (Pearson correlation coefficient ranging from 0.52 to 0.96 across all the studied areas, monkeys and sessions). More importantly, predictive information in the examined LFP features was mostly redundant to the predictive information available in kinematics. In other words, models combining both LFP features and kinematics typically improved only marginally over models using only kinematics in the studied 3-D reach and grasp task. These results should not be dismissed as overfitting artifacts since they were obtained under well controlled L2 regularization aiming to preserve generalization of models with larger number of parameters. Furthermore, in the few cases for which LFP features seemed to add predictive information with respect to kinematics, this information turned out to be redundant to the information available in short term correlations in the intrinsic spiking history. Overall, our findings suggest that multiband LFP oscillations in motor cortex of alert behaving primates, although predictive of single-neuron spiking during movement execution, are primarily related to collective dynamics controlling aspects of motor output (e.g., kinematics) rather than other potential ongoing dynamics not directly related to the task (e.g., arousal levels).

Several previous studies have looked at the relationship between single-neuron spiking and features of LFP oscillations, mostly in sensory cortices and during anesthesia (e.g., Haslinger et al., 2006; Kelly et al., 2010). Recent work by Ecker et al. (2014) has shown that previously reported high correlations between neuronal pairs and strong phase locking to ongoing LFPs in primary visual cortex during stimulation were highly dependent on the anesthesia state, with neuronal ensemble spiking becoming much more asynchronous during awake stimulation tasks. Our analysis goes beyond previous studies by examining motor cortex LFP and spiking in awake behaving non-human primates. Furthermore, to our knowledge, this is the first time that the redundancy between the information available in multiband LFP features and the information available in behavioral output (kinematics) has been systematically assessed in motor cortex. It remains to be seen how much of the residual variability is inherent to stochastic aspects of the biophysics (e.g., noise due to synaptic failure and amplification effects during spike generations; Carandini, 2004), to other motor-related covariates (e.g., torques and muscle activations) not examined in this paper, or to network dynamics not faithfully reflected in LFP features. In the latter, it is possible that the cortical layer from which the electrode tips recorded (likely layer 5 in our data) may impact LFP predictive power. For example, LFPs recorded from layers 2/3 of motor cortex may potentially exhibit different spike prediction performance and different levels of redundancy with respect to kinematics. In addition, we note that typically recorded LFPs might not be as “localized” as previously thought (Kajikawa and Schroeder, 2011). In particular, rhythmic oscillations in electric potentials recorded intracellularly and on broad extracellular fields may share similar frequencies, and yet show very different phase-locking dynamics with respect to neuronal spiking (e.g., Harvey et al., 2009). Thus, the broader LFP spatial average might result in signals that are less predictive of single-neuron spiking and more related to population activity.

The relationship between single-neuron spiking and ongoing LFP oscillations, in particular the locking of neuronal spiking to the phase of oscillations in specific frequency bands, might be highly dependent on the neuron types (e.g., pyramidal vs. fast spiking interneurons; Buzsáki et al., 2012). Recent work by Vigneswaran et al. (2011) has demonstrated that certain types of pyramidal neurons in primary motor and premotor cortices can show features of action potential waveforms and spiking statistics that are indistinguishable from features in inhibitory interneurons. Therefore, an analysis based on such putative classification would remain highly questionable in our motor cortex data.

In our analysis, low frequency (0.3–2 Hz) and higher (>100 Hz) frequency LFP bands tended to contribute the most to prediction of neuronal spiking. The former relate to motor evoked potentials, which are known to be highly correlated with the population spiking (Bansal et al., 2012), and the latter to multiunit activity, whose movement-related modulation also reflects correlated spiking in neuronal populations. Intermediate frequency bands tended to contribute little during movement execution in this type of task. One could raise the possibility that the relationship between LFP features and single-neuron spiking in these intermediate frequency bands could be much more transient than the relationship between spiking and kinematics during movement execution. For example, beta oscillations, even during movement preparation, typically occur in transient, not sustained, events lasting a few or several cycles. Thus, one would like to build models in which spiking phase-locking should be obviously conditioned on the amplitude of the beta filtered LFPs, so that these transients can be properly captured. In this regard, we note that the neural point process models used here should capture such dependence on beta amplitude, since the log-additive form of the models allows for (nonlinear) multiplicative effects and interactions among different terms (e.g., beta amplitude and phase) in the models. We also note that, although more complex LFP features and models could potentially improve spike prediction, the same could be said about improving the predictive power of motor behavioral covariates by using more complex or a larger set of kinematic features, including for example kinetics (torques) and muscle activation covariates. We hope to be able to examine more complex LFP and motor behavior-related features in the future.

The results reported here on the redundancy between motor-cortex multiband-LFP features and motor behavior are specific to execution of motor tasks in non-human primates who were highly engaged during movement execution. Multiband LFP features also provide reliable biomarkers for broader brain states and their changes. For example, the relationship between single-neuron spiking activity and ongoing LFPs is likely to change substantially depending on anesthesia, drowsiness, resting vs. awake states, attentional and volitional states, as well as stages during motor tasks (e.g., preparation vs. execution). In this broader context, including a larger variety of neural states than examined in this study, we expect multiband LFP features will be an important independent signal to account for neuronal spiking variability not explained by stimuli or behavioral covariates.

Variability in single-neuron spiking activity has often been characterized as of two types: private and shared (e.g., Deweese and Zador, 2004; Churchland and Abbott, 2012; Litwin-Kumar and Doiron, 2012; Goris et al., 2014). Private variability is likely to reflect chaotic nonlinear dynamics in highly recurrent neuronal networks (Litwin-Kumar and Doiron, 2012). Amplification of membrane potential fluctuations by the spiking generation process (Carandini, 2004) in addition to local stochastic factors such as thermal fluctuations and synaptic failure (Faisal et al., 2008) are also important contributors. On the other hand, shared variability in neuronal ensembles is thought to evolve on slower time scales and reflect representational and computational states in neuronal networks (Churchland and Abbott, 2012; Litwin-Kumar and Doiron, 2012). The examined fluctuations in multiband LFP oscillations seem primarily to be related to this shared variability. Multiband oscillatory LFP activity results in large part from coherent or shared dynamics in neuronal networks. In addition, features in these oscillations that are predictive of single-neuron spiking seemed mostly redundant to parameters in motor behavior. Overall, our finding was that information in the examined multiband LFP features directly relates to these shared representational and computational dynamics across neural populations in motor cortex. Single-neuron activity in motor cortex populations has been shown to be dominated by latent low-dimensional collective dynamics (Truccolo et al., 2010; Churchland et al., 2012). We hope in the future to investigate the relationship between multiband oscillatory LFP activity, in particular slow fluctuations, and latent low-dimensional rhythmic dynamics (Churchland et al., 2012) in motor cortex.

### Conflict of interest statement

The authors declare that the research was conducted in the absence of any commercial or financial relationships that could be construed as a potential conflict of interest.
